# St. Bartholomew's Hospital

**Published:** 1905-02-04

**Authors:** 


### HOSPITAL ADMINISTRATION.

### CONSTRUCTION AND ECONOMICS.

### ST. BARTHOLOMEW'S HOSPITAL.

THE appointment of Lord Ludlow as treasurer of St. Bartholomew's Hospital may or may not prove important not only to that institution but to the London hospitals generally. Lord Ludlow has the reputation of being a man of energy and capacity, and his experience as a barrister and a member of the London County Council should prove useful to him in the administrative work for which he has now become responsible. Whether his appointment will save St. Bartholomew's Hospital from the decadence with which it is threatened, must largely depend upon Lord Ludlow himself. We hope he may have accepted office with the full determination to take his own line, and, by asserting his independence, to at once secure the co-operation of the strongest and most representative men he can attract to the work for the office of almoner and as members of the House Committee. If Lord Ludlow refuses to have his hands tied by any commitments of his predecessors in office, and decides to take an independent line at the outset, he will have a great opportunity to add to his reputation and make St. Bartholomew's Hospital worthy in every way of its great traditions.

The position of affairs at the present moment so far as the governors are concerned is most unsatisfactory. Two of the most independent and representative men amongst them accepted seats on the House Committee in good faith, resolved to devote themselves to the work of the hospital. They found, however, as both have publicly stated, that the management has fallen into the hands of a small clique who act together and prevent the introduction of reforms, and the enforcement of efficiency. These gentlemen, Mr. Alderman Guthrie, M.P., and Mr. Andrew Motion, have both recently resigned their seats on the House Committee in consequence, feeling that it is a waste of time to attend meetings where everything is cut and dried, and where independent discussion is stifled or resented. They have publicly protested against this state of unreason and prejudice. They were anxious for further information of the grounds for granting so large a pension to the late clerk; and with other governors they objected to the appointment of his son to a new office which was not advertised, and to the advertisement for a clerk during the interregnum before the appointment of a new treasurer. The latter step committed the governors to the appointment of a clerk at a relatively low salary. Indignation has been excited too by the announcement at the Mansion House meeting of a sum of money as promised or given, which was far in excess of the actual sum in hand. In the last case the error was attributed to a wrong casting, but no public correction was made, and the public was left under the impression that the large sum announced at the meeting was correct. None of these proceedings are calculated to win public confidence.

There is a general feeling too that the present policy which is apparently to rest content with the old wards as they stand and to expend about a quarter of a million pounds upon a new out-patient department, nursing home and a pathological block is indefensible. This policy, if it can be dignified by such a title, of the clique in possession of St. Bartholomew's Hospital has brought great discredit upon its administration, and has alienated public sympathy from the hospital. If anything effective is to be done to save the position the new treasurer must act with promptness and decision. The real question to be determined is whether St. Bartholomew's Hospital shall be administered upon public lines as a public institution, or whether its administration shall be confined to a few gentlemen who have practically monopolised the control, and who have shown such remarkable incapacity and want of judgment as to completely alienate outside sympathy. There are some 300 governors, and we have little doubt that if Lord Ludlow was to request the whole of them to meet him in the grand hall of St. Bartholomew's Hospital and frankly discuss the present position of affairs, he would find that the large majority of independent governors would support him in a new policy, providing he is prepared to introduce reforms and to secure that everything connected with the administration of St. Bartholomew's shall in future be open and above board, and that no one connected with its management shall have any other object in view but to promote the best interests of the hospital, and to win public sympathy for its work. We should be glad to see the errors of the past corrected by a reform of the whole administration. There is an urgent call for the introduction of a new and better spirit free from personal aims and interests, and of a policy based upon sound business principles, which can alone secure the welfare of St. Bartholomew's Hospital and all connected with it.

Lord Ludlow has then a great opportunity. If he grasps it, St. Bartholomew's Hospital can be saved. But if the new treasurer decides to place himself in the hands of the present small clique who control its affairs and to be their obedient servant, St. Bartholomew's Hospital must gradually sink into greater and greater disrepute, until it ceases to excite any public interest. The crisis is acute at the moment. In fact the future destiny of the oldest and greatest of British hospitals must largely depend upon the action of its new treasurer during the next few months. The present position of the members of the honorary medical staff, as defined by their charge on appointment, is humiliating in fact, and opposed to the highest welfare of the hospital. This grave defect in the regulations is responsible for many evils in the past, and here the new treasurer is afforded an opportunity for the exercise of statesmanship and tact. If he boldly faces the crisis and deals with it courageously, he may count upon the united support of the public and the medical profession, as well as of the most influential and representative of the governors.

